# Document-level medical relation extraction via edge-oriented graph neural network based on document structure and external knowledge

**DOI:** 10.1186/s12911-021-01733-1

**Published:** 2021-12-30

**Authors:** Tao Li, Ying Xiong, Xiaolong Wang, Qingcai Chen, Buzhou Tang

**Affiliations:** 1 Harbin Institute of Technology, Shenzhen, China; 2 Peng Cheng Laboratory, Shenzhen, China

**Keywords:** Medical relation extraction, Graph neural network, Document structure, External knowledge

## Abstract

**Objective:**

Relation extraction (RE) is a fundamental task of natural language processing, which always draws plenty of attention from researchers, especially RE at the document-level. We aim to explore an effective novel method for document-level medical relation extraction.

**Methods:**

We propose a novel edge-oriented graph neural network based on document structure and external knowledge for document-level medical RE, called SKEoG. This network has the ability to take full advantage of document structure and external knowledge.

**Results:**

We evaluate SKEoG on two public datasets, that is, Chemical-Disease Relation (CDR) dataset and Chemical Reactions dataset (CHR) dataset, by comparing it with other state-of-the-art methods. SKEoG achieves the highest F1-score of 70.7 on the CDR dataset and F1-score of 91.4 on the CHR dataset.

**Conclusion:**

The proposed SKEoG method achieves new state-of-the-art performance. Both document structure and external knowledge can bring performance improvement in the EoG framework. Selecting proper methods for knowledge node representation is also very important.

## Background

**Fig. 1:**
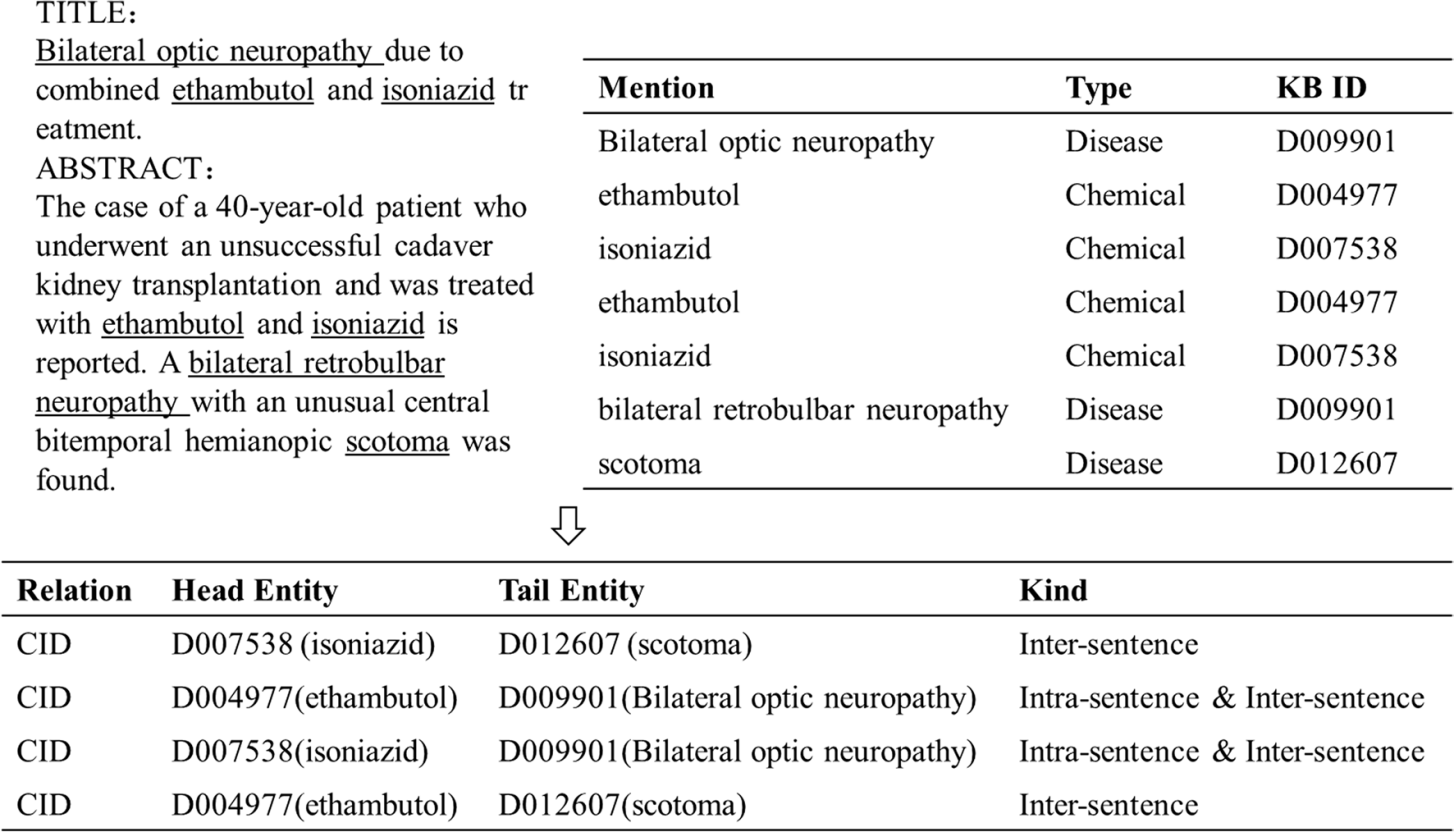
Example of document-level relation extraction

Relation extraction (RE) that extracts relations among entities in the text is a fundamental task of natural language processing (NLP). There may be two kinds of RE: (1) sentence-level RE that extracts relations in the same sentence, called intra-sentence relations; (2) document-level RE that extracts relations in the same sentence and cross sentences, and the relations cross sentences are called inter-sentence relations. Compared with sentence-level RE, document-level RE is more challenging as document-level RE needs to consider both intra-sentence relations and inter-sentence relations as a whole, as shown in Fig. 1.

In recent years, document-level RE has attracted more and more attention from researchers, and various kinds of machine learning methods have been proposed. Among these methods, multi-instance learning (MIL) first introduced by Riedel et al. [1] for document-level RE is one of the most popular. MIL models multiple entity mention pairs of the same two given entities over a document and has the ability to reduce noise in distant supervised learning [2, 3]. Although the existing MIL methods achieve considerable results, they also have some disadvantages. One disadvantage of these methods is that all entity pairs are considered individually and the implicit correlations among entities in different pairs in a document are ignored.

Graph neural networks (GNNs) that can represent the whole document and consider implicit correlations among entities in different pairs have shown great potential for document-level RE [4–10]. They may fall into two categories: (1) node-oriented GNNs [4–9]; (2) edge-oriented GNNs (denoted by EoG) [10]. Node-oriented GNNs mainly focus on node representation, while edge-oriented GNNs mainly focus on edge representation. As a relation between two entities is an instinctive edge in GNNs, edge-oriented GNNs outperformed node-oriented GNNs on document-level RE in some studies [10, 11]. In the case of EoG, document structure and external knowledge have been proved meaningful. However, there is no study to investigate them comprehensively.

In this study, based on the backbone of EoG, we propose a novel GNN to consider document structure and external knowledge for document-level RE comprehensively, called SKEoG, which is an extension of KEoG proposed in our previous study [11]. To evaluate SKEoG, we conduct experiments on two public medical datasets. Experiment results show that both document structure and external knowledge are beneficial to documental-level medical RE in the backbone of EoG, and the proposed SKEoG model achieves new state-of-the-art performance, outperforming KEoG.

## Related work

The studies most related to our work are EoG [10] and KEoG [11]. EoG is the first edge-oriented GNN for document-level RE proposed by Christopoulou et al. [10]. In EoG, information at different levels, including mention, entity and sentence, are regarded as nodes connected by five types of edges. EoG models document-level relations between entities directly and achieves much better results than node-oriented GNNs [10]. KEoG is an extension of EoG by introducing two new types of nodes regarding the document itself and knowledge concept and two new types of edges to connect the two new types of nodes. KEoG shows much better performance than EoG [11].

Inspired by KEoG [11], we propose a novel EoG that considers document structure and external knowledge comprehensively, that is SKEoG. Based on KEoG, SKEoG further introduces two new types of nodes, one regarding document structure and the other regarding external knowledge such as entity description. In this study, we use three models, that is, TransE [12], RESCAL [13] and GAT [14] to represent knowledge node based on knowledge graph respectively, and use two models, that is Doc2vec [15] and an end-to-end neural network, to represent knowledge node based on entity description.

## Methods

In this section, we first introduce RE based on document structure, and then RE based on external knowledge from two aspects: knowledge graph and entity description.

### Relation extraction based on document structure

**Fig. 2:**
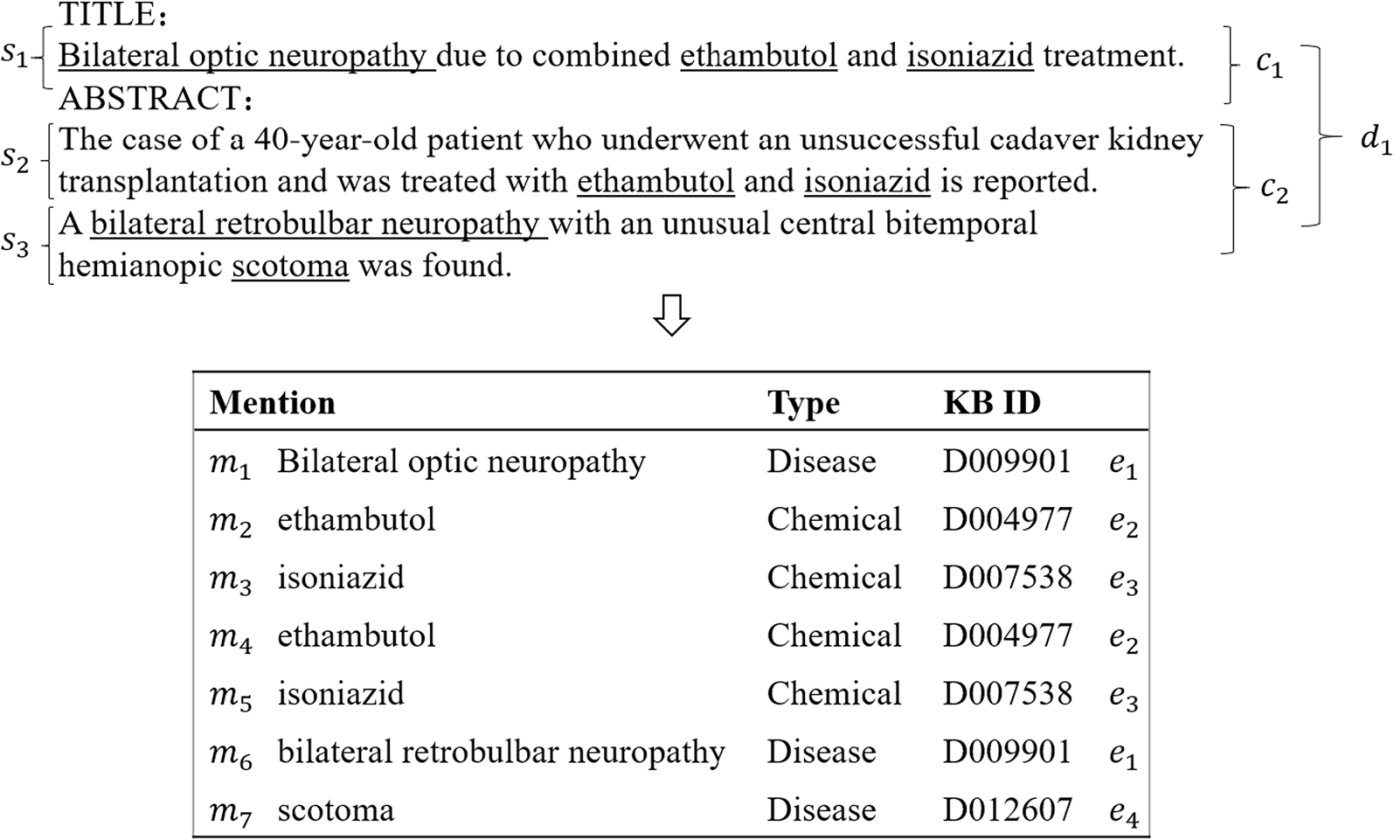
Example of document structure

A document usually has a hierarchical structure like an example, as shown in Fig. 2, where a document $d_{1}$ consists of two chapters $c_{1}$ and $c_{2}$, and each chapter contains some sentences with many entity mentions. Suppose that a sentence $s = w_{1} w_{2} \ldots w_{\left| s \right|}$, it can be represented as $H_{s}^{local} = \left[ {h_{1}^{local} ,h_{2}^{local} , \ldots ,h_{\left| s \right|}^{local} } \right]$ via an encoding layer.

In a document with |*d*| sentences $d = s_{1} ,s_{2} , \ldots ,s_{\left| d \right|}$, there are five kinds of nodes corresponding to document structure as follows:

- Mention Node (M). Each mention node $m$ is represented as $n_{m} = { }\left[ {avg_{{w_{i} \in m}} \left( {h_{i}^{local} } \right);t_{m} } \right]$, where ‘;’ denotes concatenation operation, and $t_{m}$ is an embedding to represent the node type of mention node.
- Entity Node (E). An entity $e$ is represented as $n_{e} = \left[ {avg_{m \in e} \left( {n_{m} } \right);t_{e} } \right]$, where $avg_{m \in e} \left( {n_{m} } \right)$ is the average representation of all mentions corresponding to $e$, and $t_{e}$ is an embedding to represent the node type of entity node.
- Sentence Node (S). Each sentence node $s$ is represented as $n_{s} = { }\left[ {avg_{{w_{i} \in s}} \left( {h_{i}^{local} } \right);t_{s} } \right]$, where $t_{s}$ is an embedding to represent the node type of sentence.
- Chapter Node (C). A chapter node *c* is represented by the average representation of all sentence nodes it contains and the embedding of the node type of chapter, that is, $n_{c} = \left[ {avg_{s \in c} \left( {h_{s}^{global} } \right);t_{c} } \right]$;
- Document Node (D). A document node $d$ is represented by the average representation of all chapter nodes and the embedding of the node type of document $n_{d} = \left[ {avg_{{{\text{c}} \in d}} \left( {n_{c} } \right);t_{d} } \right]$.

**Fig. 3:**
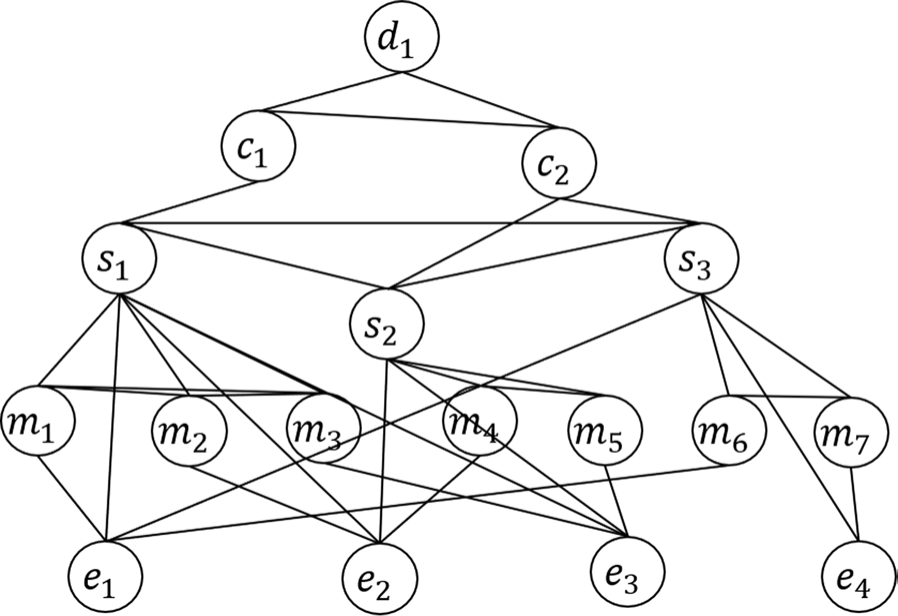
Graph to represent document structure
- Entity-Mention (ME). There is an edge between an entity mention node $m$ and the corresponding entity node $e$, that is, $e_{ME} = \left[ {n_{m} ;n_{e} } \right]$;
- Sentence-Sentence (SS). For all sentence nodes in a document, there are edges between any two sentence nodes. An SS edge is represented by $e_{SS} = \left[ {n_{{S_{i} }} ;n_{{s_{j} }} ;d\left( {s_{i} ,{\text{s}}_{j} } \right);\left| {n_{{S_{i} }} - n_{{s_{j} }} } \right|} \right](i \ne j)$, where $n_{{S_{i} }}$ and $n_{{S_{j} }}$ are the representation of $s_{{\text{i}}}$ and the representation of $s_{j}$, and $d\left( {s_{i} ,{\text{s}}_{j} } \right)$ is the representation of the relative distance between $s_{{\text{i}}}$ and $s_{j}$ measured by the number of sentences between them;
- Entity-Sentence (ES). When there is an entity mention node $m$ corresponding to an entity node $e$ in a sentence $s$, there is an edge between $e$ and $s$. The edge is represented as $e_{ES} = \left[ {n_{e} ;n_{s} } \right]$;
- Sentence-Chapter (SC). There is an edge between a sentence node $s$ and a chapter node $c$, and it is represented as $e_{SC} = \left[ {n_{s} ;n_{c} } \right]$;
- Chapter-Chapter (CC). There is an edge between two chapter nodes $c_{1}$ and $c_{2}$ in a document, and it is represented as $e_{CC} = \left[ {n_{{c_{1} }} ;n_{{c_{2} }} } \right]$;
- Chapter-Document (CD). There is an edge between a chapter node $c$ and a document node $d$, and it is represented as $e_{DC} = \left[ {n_{d} ;n_{c} } \right]$.

Given the five kinds of nodes above, we connect them with the following six kinds of edges, as shown in Fig. 3:

- Mention-Sentence (MS). When an entity mention $m$ appears in a sentence, there is an edge between the corresponding entity mention node and the sentence node $s$, and the edge is represented as $e_{MS} = \left[ {n_{m} ;n_{s} } \right]$;
- Mention-Mention (MM). When two entity mentions $m_{1}$ and $m_{2}$ appear in the same sentence $s$, there is an edge between the two corresponding entity mention nodes $n_{{m_{1} }}$ and $n_{{m_{2} }}$. The edge can be represented as $e_{MM} = \left[ {n_{{m_{1} }} ;n_{{m_{2} }} ;c_{{m_{1} m_{2} }} ;d\left( {s_{1} ,{\text{s}}_{2} } \right)} \right]$, where $d\left( {m_{1} ,{\text{m}}_{2} } \right)$ is the representation of the relative distance between the two entity mentions in the sentence, and $c_{{m_{1} m_{2} }}$ is the attention vector between the two entity mentions calculated by the following equations:
  1 $$\begin{array}{*{20}c} {\alpha_{k,i} = n_{{m_{k} }}^{T} w_{i} ,} \\ \end{array}$$
  2 $$\begin{array}{*{20}c} {a_{k,i} = \frac{{\exp \left( {\alpha_{k,i} } \right)}}{{\mathop \sum \nolimits_{{j \in \left[ {1,n} \right],j \notin m_{k} }} \exp \left( {\alpha_{k,j} } \right)}},} \\ \end{array}$$
  3 $$\begin{array}{*{20}c} {a_{i} = \frac{{a_{1,i} + a_{2,i} }}{2},} \\ \end{array}$$
  4 $$\begin{array}{*{20}c} {c_{{m_{1} ,m_{2} }} = H^{T} a,} \\ \end{array}$$
  where $k \in \left\{ {1,2} \right\}$, $a_{i}$ is the attention weight of the *i*th word in the entity mention pair <$m_{1} ,m_{2}$>, and $H \in R^{hidden\_dim \times \left| s \right|}$ is the representation of sentence $s$;

We further apply a linear transformation to all edge representations using the following equation:

5 $$\begin{array}{*{20}c} {v_{z}^{\left( 1 \right)} = {\varvec{W}}_{z} e_{z} ,} \\ \end{array}$$

where $z \in \left\{ {MS,{ }MM,ME,SS,ES,SC,CC,CD} \right\}$ and ${\varvec{W}}_{z}$ is a learnable parameter matrix.

### Relation extraction based on external knowledge

To utilize external knowledge, we regard any entity in external knowledge that also appears in text as an additional node and connect it to the corresponding entity node in text. In this paper, we introduce two kinds of knowledge nodes according to the forms of external knowledge of entities: (1) entity description and (2) knowledge graph.

**Fig. 4:**
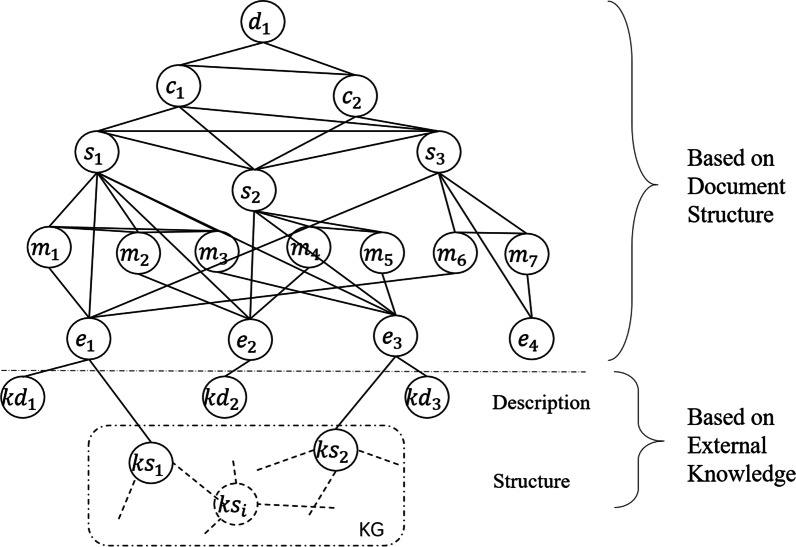
Graph based on document structure and external knowledge

Suppose that $e_{1}$, $e_{2}$ and $e_{3}$ have their external description, $e_{1}$ and $e_{3}$ exist in an external knowledge graph, the graph based on document structure as shown in Fig. 3 can be extended to the graph as shown in Fig. 4 after adding knowledge nodes, where $kd_{i}$ and $ks_{j}$ denote knowledge node based on entity description and knowledge node based on knowledge graph, respectively. In this way, we can obtain a graph that takes full advantage of external knowledge as much as possible.

### Knowledge node representation based on knowledge graph

We deploy a translation distance model, a semantic matching model and a graph model, that is, TransE [12], RESCAL [13] and GAT [14], to represent knowledge nodes based on knowledge graph respectively.

TransE assumes that any triple $\left\langle {h, r, t} \right\rangle$, where $h$ is a head entity node, $r$ is a relation, and $t$ is a tail entity node, satisfies the hypothesis of $h + r \approx t$, so as to ensure that the distance between two entity nodes is close to the representation of the relation between the two nodes. In this way, the multi-hop relation between two entities can be represented by additive transitivity, that is, if there is a relation $r_{1}$ between $h_{1}$ and $t_{1}$, a relation $r_{2}$ between $t_{1}$ and $t_{2}$, …, and a relation $r_{K}$ between $t_{K - 1}$ and $t_{K}$, there is an implicit relation between $h_{1}$ and $t_{K}$ as follows:

6 $$\begin{array}{*{20}c} {h_{1} + r_{1} + r_{2} + \cdots + r_{K} \approx t_{K} ,} \\ \end{array}$$

The max-margin function of negative sampling is used as the objective function of TransE:

7 $$\begin{array}{*{20}c} {L = \mathop \sum \limits_{{\left( {h,r,t} \right) \in \Delta }} \mathop \sum \limits_{{\left( {h^{\prime},r^{\prime},t^{\prime}} \right) \in \Delta^{\prime}}} max\left( {f_{r} \left( {h,t} \right) + \gamma - f_{{r^{\prime}}} \left( {h^{\prime},t^{\prime}} \right),0} \right),} \\ \end{array}$$

where $\left( {h,r,t} \right) \in {\Delta }$ is a true triplet, while $\left( {h^{\prime},r^{\prime},t^{\prime}} \right) \in{\Delta^{\prime}}$ is a negative triplet obtained by sampling, $f_{r} \left( {h,t} \right)$ is the score of $\left( {h,r,t} \right)$, and $\gamma > 0$ denotes the margin usually set to 1. Finally, the learned $h$ is regarded as $h_{ks}$, the knowledge node representation corresponding to node $ks$ without considering its type.

RESCAL captures the potential semantics between two entities through the bilinear function as follows:

8 $$\begin{array}{*{20}c} {f_{r} \left( {h,t} \right) = h^{T} M_{r} t,} \\ \end{array}$$

**Fig. 5:**
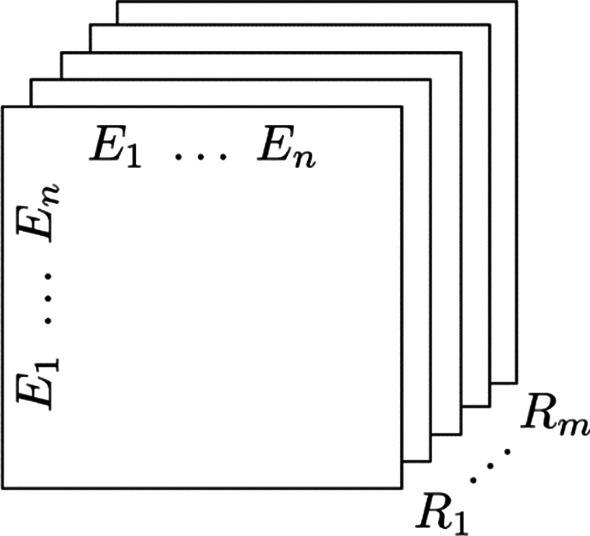
Three-dimensional tensor used to represent relation triple in RESCAL

As shown in Fig. 5, RESCAL represents relation triples as a three-dimensional tensor ${\mathcal{X}}$, where ${\mathcal{X}}_{ijk} = 1$ indicates that there is a true triplet $\left\langle {e_{i} ,r_{k} ,e_{j} } \right\rangle$. The tensor decomposition model is used to model the relationship implicitly:

9 $$\begin{array}{*{20}c} {{\mathcal{X}}_{k} \approx AR_{k} A^{T} ,\;{\text{for }}\;k = 1, \ldots ,m,} \\ \end{array}$$

where ${\mathcal{X}}_{{\text{k}}}$ is the $k$th component of ${\mathcal{X}}$, $A \in R^{n \times r}$ contains the potential representations of entities, $R_{k} \in R^{r \times r}$ is a symmetric matrix used to model the potential interactions in the $k$th relation:

10 $$\begin{array}{*{20}c} {f\left( {A,R_{k} } \right) = \frac{1}{2}\mathop \sum \limits_{i,j,k} \left( {{\mathcal{X}}_{ijk} - {\varvec{a}}_{i}^{T} R_{k} {\varvec{a}}_{j} } \right)^{2} ,} \\ \end{array}$$

where $h_{ks}$ is the component of $A$ corresponding to node $ks$.

In addition, we also represent the knowledge node $ks$ by the subgraph centered on the node using GAT.

Based on knowledge graph, a node $ks$ is represented by $n_{ks} = \left[ {h_{ks} ;t_{ks} } \right]$, where $h_{ks}$ is the representation obtained from TransE, RESCAL or GAT, and $t_{ks}$ is the embedding of the node type of knowledge graph node. The edge between an entity node $e$ and the corresponding knowledge node $k{\text{s}}$ is represented as $e_{EKS} = \left[ {n_{e} ;n_{ks} } \right]$, and it is also further transformed into $v_{EKS}^{\left( 1 \right)}$ via a linear transformation function:

11 $$\begin{array}{*{20}c} {v_{EKS}^{\left( 1 \right)} = {\varvec{W}}_{EKS} e_{EKS} ,} \\ \end{array}$$

where ${\varvec{W}}_{EKS}$ is a learnable parameter matrix.

### Knowledge node representation based on description

**Fig. 6:**
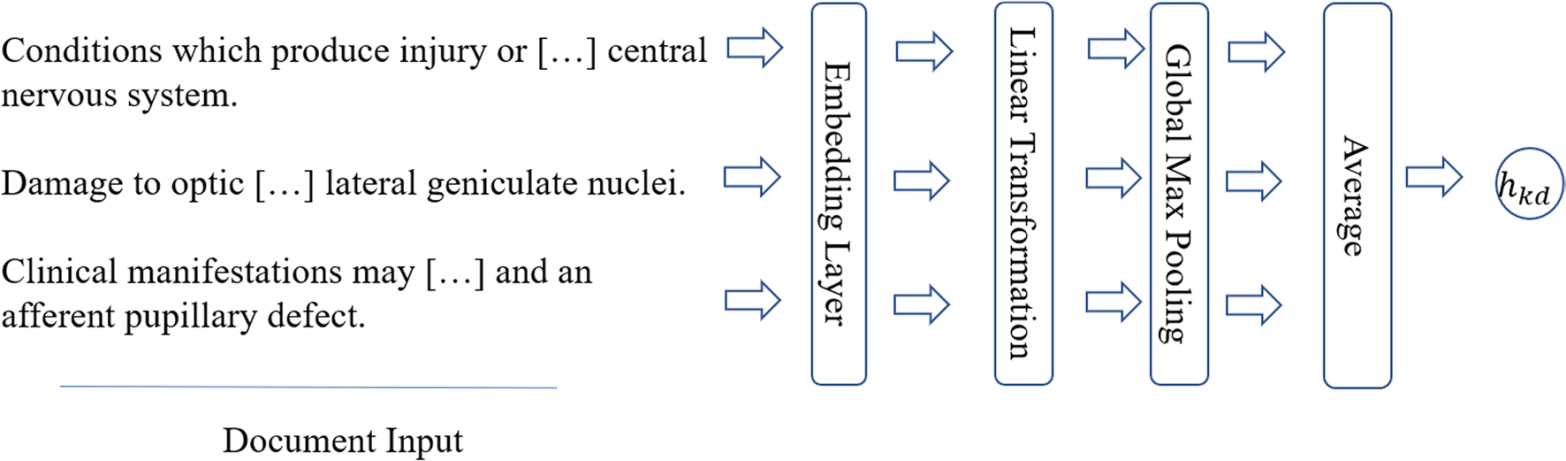
The end-to-end neural network used to represent the description of a given knowledge node

In this paper, we use the following two methods to obtain knowledge node representation based on the entity description:

1. Doc2vec [15] (also called paragraph2vec), inspired by word2vec [16] proposed by Tomas Mikolov, which can transform a sentence or a short text into a corresponding low dimensional vector representation of fixed length.
2. An end-to-end neural network, as shown in Fig. 6, which are used to encode the description text of a given knowledge node, called EMB.

Similar to knowledge node $ks$, knowledge node $kd$ based on description is represented as $n_{kd} = \left[ {h_{kd} ;t_{kd} } \right]$. The edge between $kd$ and the corresponding entity node $e$ is represented as $e_{EKD} = \left[ {n_{e} ;n_{EKD} } \right]$ and is further transformed by

12 $$\begin{array}{*{20}c} {v_{EKD}^{\left( 1 \right)} = {\varvec{W}}_{EKD} e_{EKD} ,} \\ \end{array}$$

where ${\varvec{W}}_{EKD}$ is a learnable parameter matrix.

### Inference

Following KEoG, with the help of the walk aggregation layer [17], a path between two entity nodes $i$ and $k$ of length $2l$ can be represented as

13 $$\begin{array}{*{20}c} {f\left( {v_{ik}^{\left( l \right)} ,v_{kj}^{\left( l \right)} } \right) = \sigma \left( {v_{ik}^{\left( l \right)} \odot \left( {{\varvec{W}}v_{kj}^{\left( l \right)} } \right)} \right),} \\ \end{array}$$

where $\sigma$ is the sigmoid activation function, $\odot$ is the element-wise multiplication, and ${\varvec{W}} \in {\mathbb{R}}^{{{\varvec{d}}_{{\varvec{z}}} \times {\varvec{d}}_{{\varvec{z}}} }}$ is a learnable parameter matrix used to combine two short paths of length $l$ (path between $i$ and $j$, and path between $j$ and $k$) to generate one long path of length $2l$.

All paths from node $i$ to node $k$ are aggregated to form the representation of the edge from node $i$ to node $j$ of length $2l$ as follows:

14 $$\begin{array}{*{20}c} {v_{ij}^{{\left( {2l} \right)}} = \alpha v_{ij}^{\left( l \right)} + \left( {1 - \alpha } \right)\mathop \sum \limits_{k \ne i,j} f\left( {v_{ik}^{\left( l \right)} ,v_{kj}^{\left( l \right)} } \right),} \\ \end{array}$$

where $\alpha \in \left[ {0,1} \right]$ is a linear interpolation scalar to control the contribution of edges of length $l$.

After obtaining the path representation of any entity pair of interest, we adopt the softmax function as classifier. Like in KEoG, both cross-entropy loss function and soft F-measure loss function are used as a part of the total loss function.

## Experiments

### Datasets

We conduct all experiments on the following two datasets:

- *Chemical-Disease Relation (CDR) dataset* is a dataset for document-level chemical-induced disease (CID) relation extraction, which is provided for the BioCreative V challenge [18]. It contains a training set of 500 abstracts, a development set of 500 abstracts and a test set of 500 abstracts from PubMed.
- *Chemical Reactions dataset (CHR) dataset* [9] is a dataset provided by the national text mining center (NaCTeM) of the school of computer science, University of Manchester. It contains 12,094 PubMed abstracts with their titles. Following Li et al. [11], we split the CHR dataset into a training set of 7,298 PubMed abstracts, a development set of 1,182 PubMed abstracts and a test set of 3,614 PubMed abstracts.

In this paper, MeSH^1^ and Biochem4j^2^ are used as the external knowledge of the CDR dataset and CHR dataset, respectively.

### Experimental settings

Following our previous work, we first train all models on the training set, select the best hyper-parameters on the development set, then use the same hyper-parameters retrain on the combined set of the training set and development set, and finally report the results on the test set.

For the CDR dataset, hypernym filtering is also used to ensure that only relations between hyponym entities are kept, rather than relations between rough hypernym entities. For the CHR dataset, the entities that are not in Biochem4j are removed, and the self-relations, whose head entity and tail entity are same, are removed. The statistics of the two datasets are listed in Table 1, where “#*” denotes the number of ‘*’, the numbers split by ‘/’ are the total number of pairs and the number of inter-sentence pairs.

**Table 1:** Statistics of the CDR and CHR datasets

Dataset	CDR			CHR		
	*#doc*	*#positive*	*#negative*	*#doc*	*#positive*	*#negative*
Train	500	1038/284	4202/2746	7298	19,644/6438	33,860/20816
Dev	500	1012/246	4075/2478	1182	3186/1051	5535/3425
Test	500	1066/319	4138/2593	3614	9578/2962	16,151/9708

We start with EoG that only considers document structure, called SEoG, then investigate EoG that considers both document structure and external knowledge, i.e., SKEoG, and finally compare them with other state-of-the-art methods. For convenience, we use “SKEoG(KG + KD)”, such as “SKEoG(TransE + Doc2vec)”, to denote the SKEoG model using “KG” to obtain knowledge node representation based on knowledge graph and “KD” to obtain knowledge node representation based on entity description. All word embeddings are initialized by the pre-trained PubMed word embeddings [19]. Precision (P), recall (R) and F1-score (F1) are used as measures for model performance evaluation.

## Results

We compare SEoG with other state-of-the-art methods, and the results are shown in Table 2. SEoG outperforms all other methods on the CDR and CHR test sets except KEoG(node) on the CHR dataset, which considers knowledge nodes based on the knowledge graph. To investigate the effect of the document structure presented in this paper, we further compare SEoG with its variant that does not consider chapter node, i.e., KEoG(node) without using knowledge nodes. SEoG shows much better performance than that variant (overall F1-score: 69.6 vs 67.9 on the CDR dataset, 90.4 vs 89.0 on the CHR dataset). This result indicates that the introduced chapter node is effective.

**Table 2:** Comparison results of SEoG and other different methods on the CDR and CHR test sets (%)

Dataset	Method	Overall			Intra	Inter
		*P*	*R*	*F*1	*F*1	*F*1
CDR	Gu et al. [3]	55.7	68.1	61.3	57.2	11.7
	Verga et al. [20]	55.6	70.8	62.1	–	–
	Nguyen and Verspoor [5]	57.0	68.6	62.3	–	–
	Sahu et al. [9]	52.8	66.0	58.6	–	–
	Christopoulou et al. [10]	62.1	65.2	63.6	68.2	50.9
	KEoG (node) [11]	**65.4**	71.2	68.2	71.8	58.3
	SEoG	64.5	**75.5**	**69.6**	**73.4**	**59.9**
CHR	CNN-RE [9]	81.2	87.3	84.1	–	–
	RNN-RE [9]	83.0	90.1	86.4	–	–
	Sahu et al. [9]	84.7	90.5	87.5	**–**	**–**
	KEoG (node) [11]	**89.9**	**92.6**	**91.2**	**93.4**	**86.3**
	SEoG	88.3	92.6	90.4	93.1	84.4

Bold highlight the highest result on a given dataset in our experiments

## Discussion

To investigate the effect of different knowledge node representations base on the knowledge graph for SKEoG, we compare SKEoG using different knowledge node representations with SEoG and present the results in Table 3. SKEoG using a specific knowledge node representation can achieve better performance than SEoG on both the CDR and CHR datasets. On the CDR dataset, SKEoG(TransE) achieves the highest F1-score of 70.4, while SKEoG(RESCAL) achieves the highest F1-score of 91.4 on the CHR dataset. It is a little strange that SKEoG(RESCAL) and SKEoG(GAT) even perform worse than SEoG in some cases. These results may be caused by the characteristics of different knowledge graphs used for knowledge node representation. There are only three types of relations in MeSH, and each entity has only one neighbor on average, that is, most of the existing entities and relations in MeSH can be effectively modeled by TransE, rather than RESCAL and GAT. In Biochem4j, there are nine types of relations, and each entity has three neighbors on average. The one-to-many, and many-to-one complex relations in biochem4j cannot be completely modeled by TransE, but can be modeled well by RESCAL.

**Table 3:** Effect of different knowledge node representations base on knowledge graph on the CDR and CHR test sets (%)

Dataset	Method	Overall			Intra	Inter
		*P*	*R*	*F*1	*F*1	*F*1
CDR	SEoG	64.5	75.5	69.6	73.4	59.9
	SKEoG (TransE)	65.7	75.7	**70.4**	**74.0**	**60.5**
	SKEoG (RESCAL)	**67.7**	70.4	69.0	73.0	58.4
	SKEoG (GAT)	64.7	72.5	68.4	72.6	57.6
CHR	SEoG	88.3	92.6	90.4	93.1	84.4
	SKEoG (TransE)	89.9	91.4	90.6	93.2	85.0
	SKEoG (RESCAL)	**90.6**	92.1	**91.4**	**93.6**	**86.4**
	SKEoG (GAT)	87.5	**93.6**	90.5	93.1	84.6

Bold highlight the highest result on a given dataset in our experiments

Moreover, we also investigate the effect of different knowledge node representations base on entity description for SKEoG by comparing SKEoG(TransE) using different knowledge node representations with SKEoG(TransE) on the CDR dataset. Table 4 shows the comparison results. We can see that the knowledge node representation learned by EMB can bring performance improvement by an F1-score of 0.3%. However, the knowledge node representation learned by Doc2vec hurts the performance of SKEoG(TransE). The results indicate that we should be careful to utilize the knowledge based on entity description.

**Table 4:** Effect of different knowledge node representations base on description on the CDR test sets (%)

Method	Overall			Intra	Inter
	*P*	*R*	*F*1	*F*1	*F*1
SKEoG (TransE)	65.7	75.7	70.4	74.0	60.5
SKEoG (TransE + Doc2vec)	**66.2**	72.2	69.1	73.7	56.9
SKEoG (TransE + EMB)	65.5	**76.6**	**70.7**	**74.3**	**61.3**

Bold highlight the highest result on a given dataset in our experiments

## Conclusion

We extend our previous work KEoG to SKEoG, which takes full advantage of both hierarchical document structure and external knowledge for document-level medical RE. In this study, we comprehensively investigate different methods to obtain knowledge node representation based on knowledge graph and entity description. Experimental results on two public datasets show that both document structure and external knowledge are beneficial to medical RE in the EoG framework. In the case of external knowledge, selecting proper methods for knowledge node representation is also very important.

## Data Availability

The datasets analyzed during the current study are available in the https://biocreative.bioinformatics.udel.edu/tasks/biocreative-v/track-3-cdr/ (accessed: Sep. 17, 2021) website and in the http://www.nactem.ac.uk/CHR/ (Sep. 17, 2021) website.

## Abbreviations

RE: Relation extraction
CDR: Chemical-disease relation
CHR: Chemical reactions dataset
NLP: Natural language processing
MIL: Multi-instance learning
GNNs: Graph neural networks
EoG: Edge-oriented GNNs

## Footnotes

1 ftp://ftp.nlm.nih.gov/online/mesh/2017/mesh2017.nt.

2 http://biochem4j.org/.
